# Cow’s Milk Protein Allergy in Infancy: A Risk Factor for Functional Gastrointestinal Disorders in Children?

**DOI:** 10.3390/nu10111716

**Published:** 2018-11-09

**Authors:** Licia Pensabene, Silvia Salvatore, Enza D’Auria, Francesca Parisi, Daniela Concolino, Osvaldo Borrelli, Nikhil Thapar, Annamaria Staiano, Yvan Vandenplas, Miguel Saps

**Affiliations:** 1 Department of Medical and Surgical Sciences, Pediatric Unit, University “Magna Graecia” of Catanzaro, 88100 Catanzaro, Italy; parisifrancesca01983@gmail.com (F.P.); dconcolino@unicz.it (D.C.); 2 Department of Medicine and Surgery, Section of Pediatrics, University of Insubria, 21100 Varese, Italy; silvias.varese@gmail.com; 3 Department of Pediatrics, Vittore Buzzi Children’s Hospital-University of Milan, 20154 Milan, Italy; enza.dauria@unimi.it; 4 Neurogastroenterology and Motility Unit, Department of Gastroenterology, Great Ormond Street Hospital for Children, London WC1N 3JH, UK; Osvaldo.Borrelli@gosh.nhs.uk (O.B.); Nikhil.Thapar@gosh.nhs.uk (N.T.); 5 Department of Translational Medical Science, Section of Pediatrics, University of Naples “Federico II”, 80131 Naples, Italy; staiano@unina.it; 6 KidZ Health Castle, Universitair Ziekenhuis Brussel, Vrije Universiteit Brussel, 1090 Brussels, Belgium; Yvan.Vandenplas@uzbrussel.be; 7 Division of Pediatric Gastroenterology, Hepatology and Nutrition, Holtz Children’s Hospital, University of Miami, Miller School of Medicine, Miami, FL 33136, USA; msaps@med.miami.edu

**Keywords:** CMA, allergy, hypersensitivity, FGIDs, gastrointestinal, abdominal pain

## Abstract

The role and prevalence of cow’s milk protein allergy (CMA) in functional gastrointestinal disorders remains unclear. The aim of this review is to update knowledge on the relationship between CMA and functional abdominal pain disorders (FAPDs) in children. Cochrane Database and Pubmed were searched from inception using general and specific terms for CMA and functional gastrointestinal disorders. CMA is reported as a predisposing or coexisting factor in a wide range of functional gastrointestinal disorders in infants and children. Pathogenesis of both conditions is complex and multiple mechanisms including dysmotility and hypersensitivity might contribute to the clinical manifestations. Data supporting the possible role of food allergies in the pathogenesis of FAPDs are limited. CMA may predispose to early life inflammation and visceral hypersensitivity, which in turn might manifest as FAPDs. The diagnosis of either CMA or FAPDs and distinction between them is challenging because of nonspecific and overlapping symptoms. Lack of accurate allergy tests in non-IgE (immunoglobulin E) mediated cases is also problematic. Oral food challenge, following an elimination diet, should be performed to diagnose a suspected non-IgE CMA allergy in children with FAPDs. In the management of FAPDs, an elimination diet should be considered for a limited period to verify if the symptoms improve or resolve.

## 1. Introduction

Cow’s milk (CM) allergy (CMA) is one of the most common food allergies in infants and young children with a prevalence of 2–5% [1,2,3,4,5,6]. Food allergy is defined as an adverse health effect related to the exposure to a given food, arising from specific immunoglobulin (Ig)E mediated, non-IgE mediated (cellular mediated), or mixed [7,8] immune responses. 

The major milk allergens are whey proteins (ß-lactoglobulin being the most abundant) and caseins [9]. Caseins consists of several isoforms: α s1-casein, α s-2 casein, β casein, and k-casein. Previous studies over the past few decades have suggested that casein may be the major cow’s milk protein toward which reactions might occur. Moreover, patients with persistent CMA showed IgE reactivity towards casein epitopes, such as α s1 and β casein, compared to those whose developed clinical tolerance [10]. More recently, it has been shown that circulating casein-specific T cells (particularly α s1 and β casein) were the most prevalent in children suffering from CMA, compared to non-CMA subjects [11,12], suggesting the possible utility of T-cell responses as a promising tool to improve CMA diagnosis, which actually still relies on oral food challenge.

There is also emerging evidence for a different pathogenetic role of the genetic types of β caseins, for example, A1 and A2 in the development of gastrointestinal symptoms in humans [13].

The majority of patients with IgE CMA are sensitized to more than one CM allergen, with a great variability in the specificity and intensity of the IgE responses. Molecular-based allergy diagnosis allows to associate each patient with a specific immunoreactive profile and to identify different CMA phenotypes [14].

CMA may be considered a predisposing or comorbid disease in patients with persisting functional gastrointestinal disorders (FGIDs), including functional abdominal pain disorders (FAPDs). FGIDs are common disorders with an estimated worldwide-pooled prevalence of 13%, which increases to up to 40% of the population in certain areas [15,16,17,18,19]. FGIDs are defined as chronic or recurrent gastrointestinal (GI) symptoms that are not explained by structural or biochemical abnormalities or that after appropriate medical evaluation cannot be attributed to another medical condition [20].

CMA induces a diverse range of symptoms of variable intensity in infants with ‘‘immediate’’ (early) and/or ‘‘delayed’’ (late) reactions. Immediate reactions usually occur from minutes up to 2 h after the ingestion of the allergen in the cases that are IgE mediated, and anaphylaxis represents the most severe clinical manifestation of IgE-mediated CMA. Conversely, in cases of non-IgE (cellular) mediated immune mechanisms, the reactions to the CM proteins are delayed and may manifest up to 48 h or even one week following the ingestion, making its diagnosis difficult to demonstrate. Moreover, there are no specific symptoms or biomarkers for non-IgE (cellular) mediated reactions. Combinations of immediate and delayed manifestations to the same allergen may occur in the same patient [8]. Although CMA may sometimes be transient and benign, as is the case for non-IgE-mediated CMA, it may overlap with or predispose to FGIDs. IgE-mediated CMA often persists into school age and is a risk factor for other atopic diseases [2].

In the present review, we focus on both CMA-related GI symptoms and CMA as a predisposing condition to subsequent FAPDs, defined as FGIDs with abdominal pain as a driving symptom**.**

## 2. Methods

We searched the Cochrane Database and PubMed from inception to 31 August 2018, using the following Keywords: “food-hypersensitivity”, “dietary protein proctocolitis”, “dietary protein enteropathy”, “neurogastroenterology”, “colic”, “constipation”, “abdominal pain”, “functional abdominal pain”, “irritable bowel syndrome”, “IBS”, “functional gastrointestinal disorders”, “gastroesophageal reflux”, “vomit”, “functional dyspepsia” and “cow’s milk allergy”, “cow’s milk protein allergy”, “food allergies”. Limits related to age (children, aged 0–18 years) and languages (English) were introduced. Intervention-controlled trials, reviews, guidelines, and meta-analyses on CMA were considered. Additional strategies for retrieving studies comprised the reference lists of review articles and included studies.

## 3. Association between Cow’s Milk Allergy and Gastrointestinal Disorders in Infants and Children

CMA may involve different organs and systems, most frequently the skin and the GI tract, followed by the respiratory tract [8,21]. GI symptoms include oral and perioral swelling, dysphagia, and food impaction (impaired esophageal motility) [22], vomiting, regurgitation, dyspepsia, early satiety and food refusal, failure to thrive [23], diarrhea (with or without malabsorption or protein loss due to enteropathy), rectal bleeding [24], abdominal pain, severe colic [25], and persistent constipation [26]. However, the clinical diagnosis is sometimes difficult, as signs/symptoms such as regurgitation and colicky crying may also occur in more than 50% of healthy infants [15]. Thus, it is important to make a correct CMA diagnosis to avoid unnecessary exclusion diets.

There are currently no laboratory tests available that can accurately and specifically diagnose GI-CMA mainly because of the lack of a reliable test for non-IgE reactions. Skin prick testing and allergen-specific IgE measurements only concur with IgE-mediated allergy. However, children with GI manifestations of CMA are more likely to have a non-IgE mechanism, compared to patients with skin manifestations; thus, a negative allergy test result does not exclude CMA [27,28]. In most cases, an oral challenge test is necessary in order to confirm an adverse reaction to CM protein (CMP) [29]. At present, the avoidance of CMP is the only management option for relieving or reducing the symptoms of CMA [7,8]. In infants with CMA, extensive hydrolyzed or elementary formula in non-breast-fed infants should be considered according to the severity of reactions [30,31,32]. In breast-fed infants, CM avoidance in maternal diet may reduce infant symptoms related to CMA, as in severe colicky behavior. Tolerance to CMP is often acquired in the first years of life [8]; hence, re-evaluation and reintroduction of CMP should be considered after 2, 6, or 12 months of diet, according to initial manifestations and allergy tests, in order to avoid a prolonged unnecessary diet [33].

In 2012, Iacovou et al. assessed the effect of a CMP elimination diet on colicky symptoms in a systematic review [34]. Based on the results of eleven randomized controlled trials (RCTs), considered of good quality, evaluating the effect of extensively hydrolyzed formulas or amino-acid-based formulas, and of two previous separate reviews [35,36], breast-fed colic infants seemed to benefit from a maternal low-allergen diet and formula-fed infants from the use of hydrolyzed formulas.

However, as data on reintroduction of CMP is missing in most of these studies, Lucassen et al. have drawn different conclusions [35]. In another systematic review [37], the authors found no conclusive evidence on the effectiveness of CMP exclusion on infant colic. Thus, the association of infant colic with CMA is far from unambiguous and needs to be further investigated.

The role of crying and pain due to CMA in infants with regurgitation and gastroesophageal reflux (GER) is also a controversial issue. Persisting regurgitation could be a nonspecific symptom of CMA in infants; irritability, crying, pain, sleep and feeding disturbance, and respiratory symptoms may occur both in CMA and primary and secondary (to CMA) GER [38]. CMA has been reported in up to half of infants presenting with persisting GER [39,40]. In a proportion of cases, GER is not only CMA-associated but also CMA-induced. Suspicion of CMA increases, especially in atopic families, as well as if the children present symptoms involving more than one organ system [23,39,41]. Other manifestations of CMA, like atopic dermatitis, rectal bleeding, or signs of malabsorption (such as diarrhea and failure to thrive), may reinforce the diagnosis, but are not necessarily present. Allergy testing (specific IgE or a positive skin prick test) are positive in only 30–40% of infants with CMA proven by a double-blind placebo-controlled challenge [28,42]. A typical pH-monitoring pattern, characterized by a progressive, slow decrease in esophageal pH between feedings, has been suggested by some authors, but not confirmed by others. As a consequence, if CMA is suspected, an elimination diet for 2–4 weeks is the recommended intervention and an oral challenge should be scheduled in “responder” infants to prove the diagnosis and, later, to identify the ones who acquire tolerance to CMP. Forty years ago, Buisseret reported the presence of vomiting, colic, difficult feeding in infants, growth retardation, psychological disturbance, and diarrhea in 79 children with CMA [33,43].

Later, the association between CMA and GER was reported in 15–42% of infants with symptoms suggestive of both conditions [26,38,40,44,45,46,47,48]. Enteropathy was found in 20% of recurrent vomiting infants [49].

The intestinal permeability test resulted >95% accurate in identifying CMA in 25 chronic vomiting infants [44]. However, intestinal permeability studies are not easily performed in most hospitals, are unspecific for CMA, and are of limited sensitivity in cases without enteropathy [50].

Several studies demonstrated that 2–100% of infants with GER symptoms who were not responsive to reflux treatment had clinical improvement on a CM-free diet and relapsed on oral challenge [38,51,52,53,54]. In 19 infants with irritability and vomiting attributed to GER (with esophagitis in 9) that persisted despite extensive hydrolyzed formula and antireflux medications, symptoms remitted in all patients within two weeks of starting aminoacid formula. After 2–12 months, a double-blind placebo-controlled challenge (DBPCC) showed that 12 infants were still intolerant to other formulas [55].

Common allergic tests and the family or patient’s medical history of allergy were not always positive and not highly predictive of response to a CM-free diet (CMFD). CMA-related GER seems limited to the very young age groups. In older children, and mainly where a family history of allergy is present, CMA symptoms are likely to evolve in cutaneous (atopic dermatitis), respiratory (wheezing, asthma, rhinitis), or lower motility disturbance (constipation) [56].

CM protein and Beta-lactoglobulin IgG antibodies have been proposed to differentiate patients with CMA–GER from GER unrelated to CMA, but with conflicting results [38]. Bradygastria and tachygastria were found to be more frequent in patients with CMA than in GER or normal children [53]*.* In CMA, gastric dysrhythmia may cause delayed gastric emptying with vomiting and pain [57] and increased of nonacid GER [58]*.*

The prevalence of CMA is difficult to determine because most of the affected infants have negative (or non-IgE) allergic tests. Conversely, data on the efficacy of a CM elimination diet are limited by physiological improvement (with time), often lack of oral food challenge, to confirm the diagnosis and effect of hydrolyzed formulas in gastric emptying. The National Institute for Health and Care Excellence (NICE) guidelines on GER suggest that the likelihood of CMA is increased in presence of regurgitation associated with chronic diarrhea, bloody stools, other atopic manifestations (i.e., eczema), or positive family history for allergy [59]. 

In the European Society for Paediatric Gastroenterology, Hepatology and Nutrition (ESPGHAN) guidelines on allergy, [8] the diagnosis of CMA is likely if regurgitations are frequent and other unexplained symptoms involving at least two different systems are present. The diagnosis of CMA needs to be confirmed or excluded with an elimination diet, lasting 2–4 weeks, followed by food challenge. In the recent review of Vandenplas, the authors proposed a new clinical score (CoMISS) to identify infants with regurgitation and associated CM symptoms. However, this clinical score has not as yet been validated [60].

CMA was first suggested as a cause of constipation in 1978 [43]. In an open study published in 1995, Iacono et al. [61] reported that 21 (78%) out of 27 children affected by chronic constipation improved after a CMP elimination diet. These early findings were later confirmed by prospective studies, although different response rates were identified by other authors [2,26,45,62,63,64,65] and the causal association between constipation and food allergy is not universally accepted [66,67].

The cause of constipation induced by CMA could be the result of increased resting anal sphincter pressure and an abnormal relaxation of the anal canal related to the presence of allergic inflammation of the rectal mucosa (characterized by an increased eosinophil and mast cell infiltration at rectal biopsy) [63]. Both the inflammatory reaction and the motility abnormalities disappear after a CMP elimination diet [63] It has been also suggested that abdominal and defecation pain in CMA-associated constipation might be caused by visceral allodynia, which is characterised by an abnormal perception of physiological stimuli, such as intestinal distention and peristalsis [68]. However, the pathophysiology for the association between CMA and constipation is still being debated [69,70].

## 4. Functional Abdominal Pain/Irritable Bowel Syndrome

FAPDs [20] define a group of FGIDs with pain as the driving symptom. Within this group, four subgroups have been defined: Irritable bowel syndrome (IBS), functional dyspepsia, functional abdominal pain-not otherwise specified (FAP-NOS), and abdominal migraine [71] with the first three diagnostic categories being more prevalent than the latter.

IBS is the most common FAPD, with most of the studies worldwide showing that 4–7% of all school children qualify for this diagnosis [72,73,74,75,76,77,78].

There has been longstanding interest regarding the possible role of food allergies in IBS, but data supporting this association are limited. Nevertheless, with the elevated prevalence of food allergies and FGIDs, it is likely that patients might have allergies or hypersensitivity without a causal relationship in all cases [9]. Although diet has traditionally been assigned a relatively minor role in the pathogenesis of IBS, 50% of patients with IBS report postprandial exacerbations of symptoms, either as a direct or deferred reaction [79,80,81]. However, a CM-free diet may ameliorate symptoms that are not exclusively related to the absence of CM protein, but through the absence of lactose (hence decreasing fermentation, distension, bloating, and diarrhea), reduced fat, and different protein source or size (accelerating gastric emptying), all mechanisms that do not involve allergic or immune-mediated responses. However, in some cases, the perception of the patients that CM triggers (and its elimination relieves) symptoms is suggestive of CMA. As IgE-based allergy tests are often negative, the real prevalence of CMA is unclear even after performing an oral challenge, as the reintroduction of nutrients may re-exacerbate symptoms independently of the mechanism. 

In the overall population, food allergies are reported in 12% of children, whereas the true prevalence is only 3% [7]. Among patients with FAP/IBS, a similar overestimation of food allergies and intolerances can be observed. The majority of patients with IBS/FAPDs develop symptoms after eating [82], amplifying the idea that certain foods trigger their symptoms. Traditionally, clinical experience indicates that families of children with FAP/IBS have come to the consultation suspecting milk intolerances or allergies in their child. In 2004, Kokkonen et al. [83], conducted a population study in Finland and almost half of the mothers with children ages 10 to 11 years experiencing frequent GI complaints reported that these symptoms were related to milk, and most of them avoided mild products. However, only 14% of those with GI symptoms were found to have CMA or lactose intolerance. Thus, two-thirds of the children who avoided milk did not have CMA or lactose intolerance. In 2011, Gijsbers et al. [84] conducted a study of 220 children with FAP (4–16 years old) and described that 20% of children reported food intolerances, but only 2.3% of them had a confirmed food allergy. In an Italian study, Grazioli et al. [85], showed that 70% of children (mean age of 4 years old) reported IBS symptoms with meals, but in only 17% of these children could a food allergy be diagnosed. These data suggest that a food allergy/intolerance can exist in conjunction with FAP/IBS, but it is unlikely the sole source of the symptoms [86].

In functional dyspepsia, 10 atopic children showed a significant different pattern of gastric motility (at electrogastrography) during oral challenge with CMP compared to 9 normal controls. Early-onset neuroimmune interactions were associated with rapid disturbance of gastric myoelectrical activity and dyspeptic symptoms [87].

Therefore, the diagnosis of either CMA or FAPDs and distinction between them is challenging because of nonspecific and overlapping symptoms. CMA may be associated with FGIDs or may manifest symptoms mimicking FGIDs. The GI symptoms associated with FAPs and IBS can also manifest in cases of food allergies and intolerances. Since it is not possible to exclude conditions under which both pathologies coexist, elucidating the pathogenesis and pathophysiology behind the patient’s symptoms may be challenging [88], especially in non-IgE mediated reactions.

## 5. Cow’s Milk Allergy and Functional Gastrointestinal Disorders: Focus on Pathogenesis

In non-IgE CMA, there is evidence of pronounced T-cell-mediated inflammatory reactions, causing increased gut permeability, which in turn allows a further activation of antigen-specific T-cells and subsequent proinflammatory cytokine production. In humans, there is emerging evidence for the proinflammatory role of A-1 β casein, probably mediated by the µ-opioid peptide BMC-7, which is released during digestion from the A1 variant of β-casein, but not from the A2 variant. It appears that peptide BCM-7 induces T-cell-mediated immune response and alterations to gut motility and transit time [13]. However, the exact pathogenetic mechanisms of non-IgE GI food allergies are still not fully known [89]. Similarly, the pathophysiology of FGIDs [16] is still not completely elucidated [90].

The new definition approved by the Rome IV committee and reflective of current scientific knowledge state that “FGIDs are the result of any combination of: Motility disturbance, visceral hypersensitivity, altered mucosal and immune function, altered gut microbiota, and altered central nervous system processing” [91].

**Figure 1:**
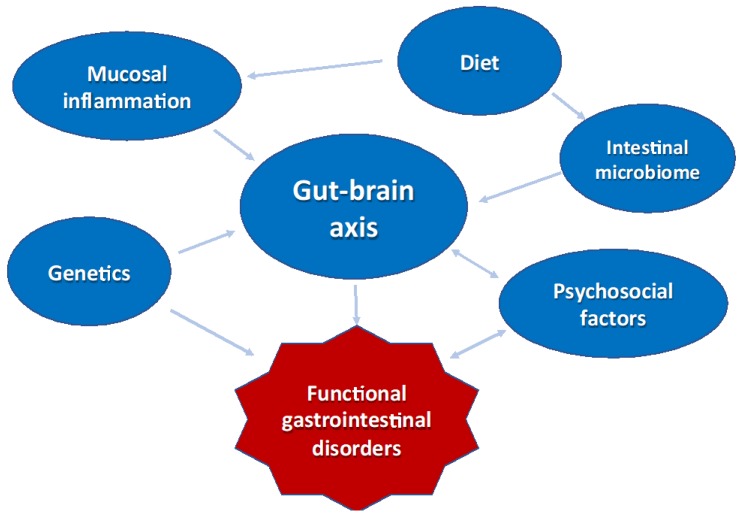
The pathophysiology of functional gastrointestinal disorders (FGIDs) is multifactorial.

Clearly, the pathophysiology of FGIDs is multifactorial (Figure 1). Genetic predisposition, impaired pain regulatory systems, sensory input (e.g., tissue damage, intestinal distension), psychological vulnerability, coping style, (family) stress, early life events, and environmental factors may all play a role in the etiology of these disorders [90]. A biopsychosocial model has also been advocated in FGIDs and concerns not only with disease, involving abnormality of the structure and/or function of organs and tissues (physical component), but also with illness, a patient’s subjective sense of feeling unwell, suffering or disability (psychological component). Both genetics and early life experiences may influence an individual’s susceptibility to copying style and FGIDs [92].

Early in life, the intestine is characterized by an immature immune system, altered intestinal permeability, and a delicate temporal window of microbiotic development, with complex interactions with the host [93,94]. Noxious stimuli in early stages may lead to the development of long-term gastrointestinal hyperalgesia through various putative mechanisms, including sensitization of primary sensory or spinal neurons, altered stress response, and/or impaired descending inhibitory control [95].

GI inflammation is considered a risk factor for the development of FAPDs. It has been shown that the development of CMP-related allergic proctocolitis [2] in the first months of life might trigger persistent digestive symptoms, particularly IBS. Other sources of inflammation, such as infectious gastroenteritis, may also trigger FGIDs (postinfectious FAPDs, particularly IBS), which can last for months to several years [41,42,96,97,98,99]. GI inflammation may also lead to visceral hypersensitivity through increased mucosal membrane permeability to antigens via alteration of tight junctions [100], increased cytokines release [101], altered mucosal immune function, microbiota [102], and receptor sensitivity in the gut mucosa and myenteric plexus. Thus, visceral hyperalgesia may result from the interaction of multiple factors, such as early adverse life events, sensitizing biological (distension, inflammation due to infection or allergy, and motility disorders) and psychosocial factors, including stressful events superimposed on a background of genetic predisposition [20]. Histological findings associated with CMA include the presence of cellular infiltrates and marked increase in eosinophils in the mucosa and submucosa with involvement of even deeper muscular layers in some cases [103,104].

Studies have linked the presence of T helper 2-associated eosinophilic inflammatory response to GI allergic hypersensitivity and gastric dysmotility [105,106]. Eosinophil granule major basic protein (MBP) decreases epithelial colonic barrier function [107]. Increased intestinal permeability has been associated with both CMA and the pathogenesis of FGIDs [108]. A study [109] has shown higher colonic permeability and GI inflammation in children with functional abdominal pain and IBS than in healthy controls. It is likely that in patients with CMA, the detrimental effect of cellular infiltration and their products on visceral nerve fibers is facilitated by the increased permeability. Sensitization of the corresponding spinal segments may result in further amplification of afferent input. The combined effect of these factors may explain the presence of short- and long-term alterations in sensation and motor function that was found in this study.

The mechanisms of immune responses to specific CMP in GI and non-IgE CMA are likely to be multiple, with more than one pathway involved [89]. Although few studies have been conducted on the role of diet in IBS, recent research has suggested that an allergy or hypersensitivity to certain foods may prompt the onset of and/or increase the severity of symptoms through immune activation [80]. Food allergy, traditionally denoted by an activation of immunoglobulin (Ig) E-mediated antibodies to a food protein, has not been linked convincingly to IBS pathogenesis, although patients with IBS have been shown to have a higher incidence of atopy [110,111,112]. Others have suggested a role for IgG-mediated immune reactions. Two studies, conducted on adult patients (>18 years), have demonstrated that when patients with IBS were given an exclusion diet to avoid foods that were shown to promote elevated IgG antibodies (such as milk, eggs, cheese, wheat, rice, potatoes, chicken, beef, pork, lamb, soya bean, fish, shrimps, yeast, tomatoes, and peanuts), a significant decline in symptoms and a corresponding improvement in rectal function were reported [113,114]. Another 12-week study conducted on adult patients, who excluded specific IgG-associated foods, resulted in a significant decrease in abdominal pain, abdominal distension, and diarrhea in patients with IBS with diarrhea (IBS-D), compared to a healthy control group [115]. However, doubt remains about the role of IgG in IBS. Zuo and colleagues found no significant relationship between IgG antibodies and symptom intensity [116], and studies demonstrating positive results have been criticized on the basis of study populations [117]. Thus, further studies on the relevance of IgG antibodies to IBS symptoms are required to confirm a tentative link [80].

## 6. Cow’s Milk Allergy as Risk Factor for Development of Functional Gastrointestinal Disorders 

Early-life allergic inflammatory triggers, especially if prolonged, may induce later digestive symptoms meeting the criteria for FGIDs, supporting the concept of “post-inflammatory” FGIDs.

To date, few epidemiological studies have evaluated the association between preschool CMA and subsequent risk of developing FGIDs later in childhood (Table 1). Saps et al. [68] conducted a hospital-based case-control study including 52 subjects between 4 and 18 years of age who were diagnosed with cow’s milk protein hypersensitivity within the first year of life; fifty-three healthy siblings of the same age were selected as controls. Twenty-three of the 52 study subjects (44.2%) reported GI symptoms that included abdominal pain, constipation, or diarrhea, compared to 11 of the 53 controls (20.75%) (OR 3.03, *p* = 0.01). Ten of the 52 subjects (19.2%) met the Rome III criteria for diagnosis of FGIDs (7 IBS, 2 functional dyspepsia, 1 functional abdominal pain), whereas none in the control group did. In this study, not all of the children diagnosed with CMA developed FGIDs: Possible suggested explanations include the fact that the inflammatory response and severity of CMA may vary from child to child. Another birth cohort study conducted in Sweden revealed an association between early allergic disease, including CMA, and recurrent abdominal pain at 12 years of age [118]. Both of these studies seem to confirm previous findings focusing on the association between CMA in infancy and FGIDs in childhood [119]. Di Nardo et al. [120] conducted a prospective controlled cohort study assessing the association between allergic proctocolitis and new-onset FGIDs. Sixteen of the 160 subjects (10.0%) included in this study met the Rome III criteria for FGIDs. Among the 80 patients with allergic proctocolitis, 12 (15.0%) reported FGIDs, compared to 4 of 80 (5.0%) controls (*p* = 0.035). They then found evidence suggesting that an inflammatory/allergic self-limiting disorder occurring early in life, such as allergic proctocolitis, is a risk factor for the development later in life of digestive symptoms meeting the Rome III criteria for FGIDs. This was due especially to IBS, which accounted for 66% of the new FGIDs in the allergic proctocolitis group. The prolonged release of inflammatory mediators during an early, vulnerable period of neural plasticity may lead to altered enteric nervous system hypersensitivity and dysmotility. Furthermore, they identified the duration of hematochezia as the only variable significantly associated with the development of FGIDs. This suggests that even in postinflammatory FGIDs, the severity of the acute trigger is a determinant of persistent digestive sequelae. An epidemiological study conducted in Taiwan on 11,242 children (age range: 7–18 years) with IBS evaluated the association among six early allergic conditions and subsequent IBS in childhood [121]. This study confirmed the existence of an association between food allergy and the subsequent development of IBS in childhood; food allergy was associated with the shortest time interval (2.35 years, SD¼ 1.8 years) before to IBS development.

A prospective cohort study, the Generation R Study, aiming to assess the association between the introduction of food allergens and gluten in the first year of life and the prevalence of constipation at 24 months of age [119], showed that a history of CMA in the first year of life was significantly associated with functional constipation in childhood (OR: 1.57; 95% CI: 1.04–2.36). The same authors, however, outlined the limitations of the parental report of a doctor-made CMA diagnosis and the use of Rome II criteria to define the outcome of the studies. Both of these limitations preclude drawing definitive conclusions. Therefore, data supporting the role of CMA as a risk factor for the development of FGIDs in children are limited and more studies are needed to fill this research gap.

## 7. Conclusions

There has been interest regarding the possible role of food allergies in the pathogenesis of FAPDs, but data supporting this association are limited. Multiple studies suggest that GI inflammation is a risk factor for the development of FAPDs. Inflammation of the GI mucosa may be due to an infectious episode, but also due to an allergic condition. Alterations in the brain–gut interactions are likely to underlie symptoms of chronic abdominal pain and associated GI dysfunction.

The majority of patients with IBS/FAPDs develop symptoms after eating, amplifying the idea that certain foods trigger their symptoms. CMA usually presents more than one organ manifestation and may be considered in persistent FGIDs, particularly when other allergic features occur. CMA may be associated with FGIDs or may manifest symptoms mimicking FGIDs. The GI symptoms associated with FAPs and IBS can also manifest in cases of food allergies and intolerances (e.g., lactose intolerance). These symptoms include nausea, abdominal pain, abdominal cramping, bloating, and diarrhea. Since it is not possible to exclude conditions under which both pathologies coexist, elucidating the pathogenesis and pathophysiology behind the patient’s symptoms may be challenging, especially in non-IgE mediated reactions. Oral challenge and CMP reintroduction should be planned to clarify the etiology and allow proper management.

## Figures and Tables

**Table 1:** Characteristics of pediatric studies evaluating cow’s milk allergy (CMA) as risk factor for functional gastrointestinal disorders (FGIDs).

FGIDs Disorder	Study and Patients Characteristics	Pathogenetic Mechanisms	Results	References
Abdominal pain, constipation, diarrhea	Case-control *n* = 52 subjects Age 4–18 yrs Diagnosis of CMA in the first year of life	Effect of eosinophils infiltration and their degranulation products on visceral nerve fibers; increased intestinal permeability	23/52 CMA diagnosis (44.2%) vs. 11/53 controls (20.75%) developed gastrointestinal symptoms (OR = 3.03)	[68]
Functional constipation at 24 months (defined according to Rome II criteria)	population-based prospective cohort (Generation R Study) *n* = 4651 Parental report diagnosis of CMA in the first year of life	Possible shift in features of cow ’s milk allergy over time with different clinical manifestations later in life compared to symptoms at the outset	OR: 1.57; 95% CI: 1.04–2.36 (after adjustment for major confounders)	[119]
Recurrent abdominal pain at 12 years of age	Birth cohort study *n* = 4089 children Parents-based questionnaires	Low-grade inflammation in the gut resulting in barrier defects in the gastrointestinal tract; increased colonic permeability; increased mast cell counts and increased tryptase release	2610 children with complete follow-up, 9% (*n* = 237) reported abdominal pain at 12 years	[118]
Irritable bowel syndrome (IBS)	Case-control study *n* = 11,242 children (age range: 7–18 years) vs. 44,968 age- and sex-matched control subjects; Physician-based diagnosis (Rome II criteria)	Visceral hypersensitivity and alterations in intestinal mobility; mucosal inflammation; dysregulated microbiota	Subsequent risk of IBS: 1.54 for Food Allergy (FA) (95% CI, 1.15–2.05) FA was associated with the shortest time interval (2.35 years, Standard Deviation 1.8 years) before IBS development	[121]
IBS, functional abdominal pain, constipation	prospective controlled cohort *n* = 160 10% FGIDs parental questionnaire on pediatric gastrointestinal symptoms, Rome III version	Abnormal mucosal milieu; Abnormal neuroimmune interactions via mast cell activation and nerve growth factor release; sensitizing medical factors (distention, inflammation, and motility disorders)	Among the 80 patients with allergic proctocolitis, 12 (15.0%) reported FGIDs, compared with 4 of 80 (5.0%) controls (*p* = 0.035); the OR for FGIDs in allergic proctocolitis group was 4.39 (95% CI, 1.03–18.68)	[120]
